# Genetic Characteristics of Resectable Colorectal Cancer with Pulmonary Metastasis

**DOI:** 10.1155/2022/2033876

**Published:** 2022-04-28

**Authors:** Yan-Yu Qiu, Dong Peng, Zheng-Qiang Wei, Jin-Dou Li, Yong-Jia Huang, Jian-Guo Yang, Zhi-Yang Song, Yong Cheng

**Affiliations:** ^1^Department of Gastroenterology, The First Affiliated Hospital of Chongqing Medical University, Chongqing 400016, China; ^2^Department of General Surgery, Jiangjin Central Hospital of Chongqing, Chongqing 402200, China; ^3^Department of Pharmacy, Jiangjin Central Hospital of Chongqing, Chongqing 402200, China

## Abstract

The lung is the most common extra-abdominal metastasis site of colorectal cancer (CRC). This study aimed to investigate the genetic variation of pulmonary metastases (PM) and primary tumors in resectable CRC. The clinical data of 410 patients with PM after CRC surgery and 33 paraffin-embedded tissue samples from January 2012 to July 2019 in our hospital were collected retrospectively. Next, 450-panel gene detection technologies based on next-generation sequencing (NGS) were used to analyze the changes in the gene map and the overall variation in cancer-related genes in PM and primary tumors. After quality control, 19 samples were included in the final gene analysis. The results showed that APC (89.5%), TP53 (89.5%), and KRAS (53%) were the most common mutations in PM and primary tumors, but the gene amplification variation was enriched in primary tumors (4.6% vs. 11.4%). KRAS G12D was the most common site variation of the KRAS gene in both PM and primary tumors of CRC. There was no hotspot mutation in the TP53 locus in CRC, and the TP53 mutation in the PM was consistent with that in the primary lesion. The microsatellite instability (MSI) levels of 10 patients were MSS. The mean tumor mutation burden (TMB) of the primary tumor (5.3 muts·Mb^−1^) was slightly higher than that of metastasis (5.0 muts·Mb^−1^). In our institution, the genetic characteristics of resectable PM from CRC may be highly consistent with those of the primary tumor.

## 1. Introduction

Colorectal cancer (CRC) is the second most common cause of cancer death in the United States [1, 2]. Pulmonary metastases (PM) are the most common extra-abdominal metastasis site of CRC [3]. Compared with colon cancer, rectal cancer has a higher incidence of PM [4]. In China, the proportion of rectal cancer in CRC cases is much higher than that in Western countries [5]. Most PM patients can only receive palliative care and have a poor prognosis. Pulmonary metastasectomy may be a curative option for carefully selected patients with limited sites of the disease. However, many patients will ultimately develop an incurable disease.

Molecular targeted therapy plays an important role in the comprehensive treatment of patients with advanced or metastatic CRC [6]. Whether a patient is eligible for anti-epidermal growth factor receptor (EGFR) antibodies (such as cetuximab and panitumumab) depends on the presence of KRAS and/or NRAS mutations [7]. KRAS is a key proto-oncogene downstream of EGFR and is activated in up to 50% of sporadic metastatic CRC patients [8]. Importantly, KRAS exon 2∼4 mutations demonstrate a significantly lower response to cetuximab and panitumumab [7, 9, 10]. Some studies have also indicated that KRAS mutations may confer resistance to bevacizumab [11, 12]. The extended RAS family of oncogenes includes NRAS, with exon 2∼4 mutations occurring in 3∼5% of CRCs and similarly resulting in a lower response [13, 14]. However, due to the heterogeneity of the primary tumor, the progress of specific tumor subcloning, and disease progression, PM may exhibit some molecular characteristics that are different from those of the primary tumor [15]. Tumor heterogeneity may limit the efficacy of targeted biological therapies. Therefore, genetic analysis of PM and primary tumors can better help doctors to determine the treatment plan and provide an additional treatment option for patients who cannot undergo pulmonary metastasectomy [5].

Immunotherapy is an emerging anticancer therapy that has recently successfully treated many cancers. Self-tolerance is maintained by the immune system through checkpoints such as programmed cell death protein 1 (PD-1). The binding of ligands (programmed death ligand 1 (PD-L1) and PD-L2) to PD-1 leads to downregulation of effector functions. One mechanism by which cancer cells remain hidden from the immune response is the upregulation of PD-1/PD-L1, which is the basis for the advances seen in the use of immunotherapy in cancer [16]. TMB is currently one of the hottest biomarkers in the field of tumor therapy and can indicate the overall immune status of the tumor [17, 18]. Patients with microsatellite instability (microsatellite instability–high (MSI-H)) can benefit from immune checkpoint inhibition [19]. Therefore, by detecting tumor mutation load and microsatellite instability, it is possible to preliminarily determine whether tumor patients are suitable for immunotherapy.

Studies have confirmed that the gene mutation status of liver metastasis is highly consistent with that of the primary CRC [20]. However, there are few large-scale studies comparing genes between CRC primary tumors and PM. In addition, there is evidence that extrahepatic metastasis is more likely to show inconsistent molecular results from the primary tumor. Therefore, gene testing of PM and primary tumors is necessary and meaningful. Based on this, we used a 450-panel gene detection technology based on the next-generation sequencing technology to analyze the cancer-related genes of PM and primary tumors in the same patient and studied the comparative overall differences in gene map changes and mutations between PM and primary tumors.

## 2. Materials and Methods

### 2.1. Patient Selection

Patients with CRC who experienced radical surgical resection of both the primary tumor and PM from January 2012 to July 2019 were selected from the First Afﬁliated Hospital of Chongqing Medical University. Clinical information, including clinical and pathological characteristics, treatment details, patient outcomes, and follow-up data were collected retrospectively by electronic medical records and telephone follow-up. Ethical approval from the institutional review board was obtained (2021–047), and informed consent was acquired from all patients.

### 2.2. Sequencing Methods

Formalin-fixed and paraffin-embedded (FFPE) tissue samples of CRC patients were collected and stained with hematoxylin eosin (H&E). After being examined by pathologists, the samples were further extracted for genomic DNA. At the same time, matched peripheral blood samples were taken as controls. The QIAamp DNA FFPE Tissue Kit (Qiagen company, Germany) and QIAamp DNA Mini Kit (Qiagen company, Germany) were used to extract genomic DNA from FFPE samples and blood samples according to the standard operation procedures specified in the instructions. Quantitative analysis was performed using a Qubit® dsDNA HS Assay Kit (Invitrogen, USA) and Qubit® 3.0 fluorescent quantizer (Invitrogen, USA).

The genomic DNA was broken into ∼250 bp fragments using a Covari LE220 ultrasound interrupter (Covaris, USA). A KAPA Hyper Prep Kit (Kapa Biosystems, USA) was used to construct the genomic DNA library. Then, the library fragment size was detected by a LabChip GX Touch HT (Perkin Elmer, USA), and the library concentration was determined by a Qubit® dsDNA HS Assay Kit (Invitrogen, USA).

The probes were designed according to the target gene sites, and probes customized from Integrated DNA Technologies were used for hybridization capture. The size of the captured library was detected by a LabChip GX touch HT (Perkin Elmer, USA), and the concentration of the captured library was determined by a Qubit® dsDNA HS Assay Kit (Invitrogen, USA). The captured library was mixed together and then sequenced by an Illumina Novaseq 6000 system in 2×150 bp double-ended sequencing mode.

### 2.3. Detection of the Tumor Mutation Burden

After the tumor tissue and matched blood samples were sequenced, they were compared to the reference sequence of the human genome, and the BAM file was generated. The paired blood was used as a control for tissue samples. Paired variation analysis was performed to remove the reproductive mutation information and retain only somatic mutations. The annotation information was used to distinguish and obtain the mutation sites in the coding region. SNP sites in public databases, including dbSNP and other databases, were further removed. The driver mutation and some artifact information were also removed to obtain the final mutation site. These sites were counted and divided by the base size (Mb) of the coding region to obtain the tumor mutation burden (TMB) value.

### 2.4. Statistical Analysis

SPSS 19.0 software was used for data analysis. The statistical data were compared by the chi square test (or corrected chi square test), the measurement data were compared by *t* test, and the difference in mutation rate between primary and metastatic lesions was performed by the Wilcoxon test. The inspection level (*α*) was 0.05.

## 3. Results

### 3.1. Patient Characteristics

From 410 colorectal cancer patients with lung metastasis, 33 patients who underwent one or more resections of CRC lung metastases were selected. The general clinical data of these 33 patients are shown in Table 1. Among them, 31 cases (93.9%) had lung metastasis after CRC surgery, 2 cases (6.1%) had lung metastasis at the initial diagnosis of CRC, and 4 cases (12.1%) had liver metastasis at the same time. There were 9 cases (27.3%) of postoperative lung metastasis from colon cancer, including 5 cases of right colon cancer (15.2%), 3 cases of sigmoid colon cancer (9.1%), and 1 case of unknown colon cancer (3.0%); 24 cases (72.7%) of postoperative lung metastasis of rectal cancer, including 15 cases (62.5%) of low rectal cancer (5 cm < anal ≤10 cm), 6 cases (25%) of middle rectal cancer (5 cm∼10 cm from anus), and 3 cases (12.5%) had high rectum length (more than 10 cm from anus).

**Figure 1:**
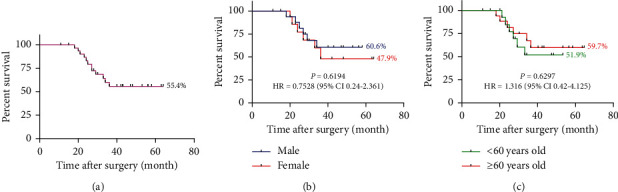
Survival analysis of 33 patients undergoing resection of pulmonary metastases. (a) Kaplan–Meier curve of the overall survival rate; comparison of overall survival between the different sex groups (b) and age groups (c) by Kaplan–Meier curves. Genetic characteristics of the primary tumor and pulmonary metastases.

At the end of the follow-up, the overall survival rate of 33 patients who underwent lung metastasis resection was approximately 55.4% (Figure 1(a)). The Kaplan–Meier curves were used to compare the overall survival rates of different sex groups. We found that the survival rate of women was slightly lower than that of men, but there was no significant difference between the two groups (Figure 1(b)). Log-rank analysis showed that sex was not associated with overall survival time (*P* > 0.05), HR = 0.7528 (95% CI 0.24–2.361). The Kaplan–Meier curve was also used to compare the overall survival rate of different age groups. We found that the survival rate of the group under 60 years of age was slightly lower than that of the group over 60 years of age, but there was no significant difference between the two groups (Figure 1(c)). Log-rank analysis showed that age was not associated with overall survival (*P* > 0.05), HR = 1.316 (95% CI 0.42–4.125). The sample size of the current study was small, with only 33 patients, and there were 7 risk factors. The multivariate models were unstable, with a limited sample size. Therefore, the Cox analysis for OS was not performed.

**Figure 2:**
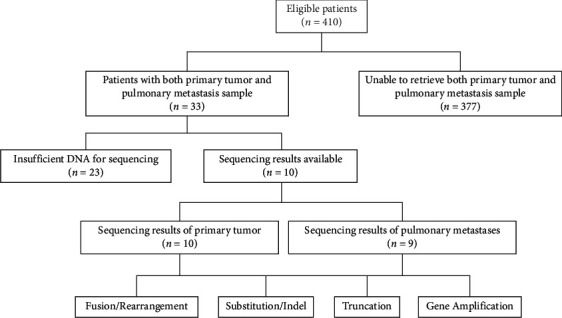
Availability of primary tumor and pulmonary metastasis samples.

Figure 2 shows the availability of primary tumor and metastasis samples and whether these samples were successfully sequenced. Primary tumor and homologous pulmonary metastasis samples were obtained from 33 patients (8.05%). Sequencing data were available for 10 primary tumors and 9 PMs from 10 patients after quality control. The variation patterns analyzed included point mutation, amplification, fusion/rearrangement, and truncation.

**Figure 3:**
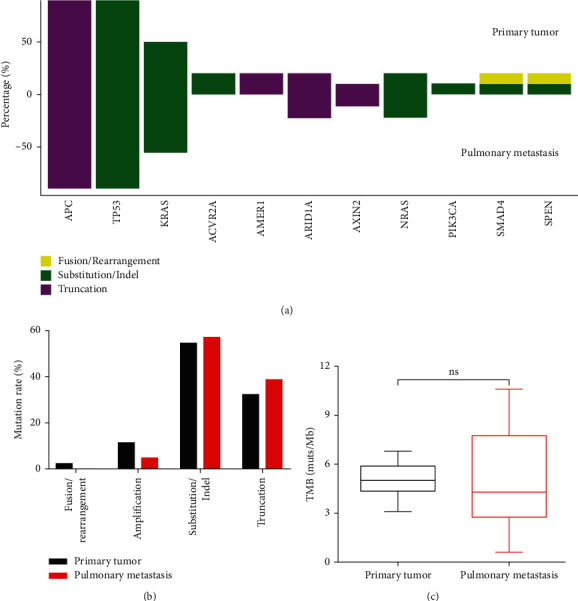
Comparison of gene mutations between primary tumors and pulmonary metastases.

APC (90%), TP53 (90%), and KRAS (50%) were the top three most frequently mutated genes in primary lesions, and APC (89%), TP53 (89%), and KRAS (56%) were the most frequently mutated genes in lung metastases (Figure 3(a)). The variation patterns of the APC/KRAS/TP53 gene in primary and metastatic foci are shown in Table 2. The most common site variation of the KRAS gene in CRC is KRAS G12D. KRAS G12D/R/V was detected in 5 cases of primary lesions, and the same site variation was detected in 4 cases of corresponding metastatic lesions. There is no hotspot mutation in the APC/TP53 locus in CRC. The APC site mutations detected in the metastatic foci had corresponding variations in the primary foci. The mutation of TP53 was identical to that of the primary tumor. Compared with metastatic foci, more gene amplification (11.4% vs. 4.6%) was detected in primary foci (Figure 3(b)). The mean tumor mutation burden (TMB) of the primary tumor (5.3 muts·Mb^−1^) was slightly higher than that of pulmonary metastasis (5.0 muts·Mb^−1^), but there was no significant difference (Figure 3(c)). Microsatellite instability (MSI) of the primary tumor and lung metastasis was also detected, and the results showed microsatellite stability (MSS).

## 4. Discussion

According to the latest research data, the incidence rate and mortality rate of CRC in men and women in the United States in 2020 will rank third among malignant tumors. [2] The liver and lung are common sites of distant metastasis of CRC. [4,21,22] There are many studies on the molecular pathology and treatment strategy of liver metastasis, but research on lung metastasis is limited. [23–26] Considering that rectal cancer patients are more likely to have lung metastasis and that the proportion of rectal cancer in CRC in China is higher than that in Western countries, [4,5] we studied the clinical and molecular characteristics of colorectal cancer patients with lung metastasis, hoping to provide a reference for the research, clinical diagnosis, and treatment of CRC with lung metastasis. In this study, rectal cancer patients accounted for a large proportion (72.7%). Blood flows from the distal parts of the rectum, surpassing the liver, and the first encountered organ is the lung. Therefore, it seems logical that we observed that rectal cancer more frequently metastasized to thoracic organs more frequently than colon cancer.

Targeted therapies are now a part of the treatment paradigm for metastatic colorectal cancer, and survival outcomes have significantly improved. [27,28] Tumor heterogeneity may limit the effectiveness of targeted therapy. Therefore, it is necessary to determine whether there is a significant molecular difference between the primary tumor and metastasis. In this study, APC, TP53, and KRAS were the first three high frequency mutation genes in lung metastases and primary foci. Compared with metastatic foci, more gene amplification (11.4% vs. 4.6%) was detected in primary foci (Figure 3(b)). The KRAS mutation may be associated with lung metastasis and prognosis in CRC patients [29–31]. Studies have shown that the KRAS mutation exists in approximately 40% of CRCs, which is related to the higher cumulative incidence of lung metastasis of CRC and is an independent predictor of lung metastasis [32]. EGFR inhibitors are not recommended for KRAS mutant patients [33]. In addition, the prognosis of lung metastasis is better than that of liver metastasis, bone metastasis, and brain metastasis [29]. APC and TP53 are genes with a high frequency of mutation in CRC, and there is no typical mutation hotspot.

RAS and RAF determinations have been performed on metastatic sites since the high concordance between RAS and RAF between primary and metastatic CRC has been demonstrated. KRAS codon 12 mutation was remarkably associated with peritoneal metastasis, liver-peritoneum metastases, and multiorgan metastases compared to all wild types [34]. The association between different mutations and clinicopathological features still needs to be explored because this might impact the translational relevance and the tailored therapy for peculiar patient subsets, as described by Brunetti et al [35].

The immune status of the tumor can reflect whether the patient can benefit from immunotherapy. The NCCN Guidelines recommend TMB as one of the biomarkers for immunotherapy in nonsmall cell lung cancer patients [33]. TMB refers to the number of somatic protein coding region point mutations, indels, and other gene mutations contained within an average of 1 Mb in the tumor genome. For patients with a TMB value higher than 10 muts·Mb^−1^, combined immunotherapy can significantly prolong their progression-free survival. [36] In this study, the mean TMB of primary tumors and lung metastases was lower than 10 muts·Mb^−1^, and there was no significant difference. Microsatellite instability (MSI) refers to any change in the microsatellite length caused by insertion or deletion of a microsatellite repeat unit in tumors compared with normal tissues, resulting in the appearance of new microsatellite alleles. Patients with microsatellite instability-high (MSI-H) can benefit from immune checkpoint inhibition. The MSI status of the primary tumor and lung metastasis in this study showed microsatellite stability (MSS).

Although the development of immune checkpoint therapy has provided a new option for the treatment of colorectal cancer patients, monotherapy with immune checkpoint therapy has not achieved efficacy as would be expected [37]. One of the treatments currently being used is chemotherapy combined with immune checkpoint inhibitors [38,39]. Cytotoxic T lymphocyte antigen-4 (CTLA-4) is an inhibitory immune checkpoint, and recent studies have demonstrated that capecitabine can significantly decrease the expression of CTLA-4 in SW480 cells [37]. Studies have shown that CTLA-4 not only attenuates the antitumor immune response by being expressed on tumor-infiltrating lymphocytes but also enables tumor cells to escape the immune response by being expressed on the tumor cell surface, thereby promoting tumor growth [40,41]. Although the mechanism by which capecitabine acts on CTLA-4 is still unclear, it may be a bridge between immunotherapy and chemotherapy [37].

To eliminate the interference of many factors such as individual differences, to the experiment to the greatest extent, we specifically screened out 33 cases from 410 CRC patients with lung metastasis in order to obtain samples of the primary tumor and lung metastasis at the same time. However, the gene panel required a substantial amount of DNA, and many samples did not yield sufficient DNA for analysis. There were many reasons for this, including exhaustion of samples by previous standard-of-care analyses or use in other clinical trials and tumor regression in resection samples due to neoadjuvant chemotherapy/chemoradiotherapy. However, this study also provides important information on the feasibility of future genomic studies in similar patient cohorts.

Our study had some limitations. First, this was a single retrospective study which might cause a selection bias. Second, the sample size of the current study was small, with only 33 patients, and there were 7 risk factors. The multivariate models were unstable with a limited sample size, so the Cox analysis for OS was not performed. Therefore, a multicenter study with a large sample size should be conducted in future experiments.

In summary, the genetic characteristics of lung metastases may be highly consistent with that of the primary tumor in CRC patients at our institution. For patients with lung metastases who cannot receive surgical treatment, it may be possible to judge whether the patient can benefit from molecular targeted therapy or immunotherapy based on the genetic characteristics of the primary tumor.

## Figures and Tables

**Table 1:** General clinical data of 33 CRC patients with pulmonary metastasis.

Characteristic	Number	Percentage (%)
Total	33	
Gender		
Male	17	52
Female	16	48
Median age (interquartile range) at diagnosis, years	60 (48–67)	
Site of the primary tumor		
Cecum/ascending colon	5	15.2
Transverse colon	0	0
Sigmoid/rectosigmoid	3	9.1
Rectum	24	72.7
Colorectal (site not specified)	1	3.0
Stage at diagnosis		
I	1	3.0
II	3	9.1
III	16	48.5
IV	3	9.1
Unknown	10	30.3
Differentiation		
Well	0	0
Moderate	20	60.6
Poor	1	3
Unknown	12	36.4
Disease-free interval (months)		
>12	21	63.6
≤12	12	36.4
CEA (ng·mL^−1^)		
>5	10	30.3
≤5	23	69.7
Number of metastases		
Single	21	63.6
Multiple	12	36.4
Surgical approach		
Pulmonary wedge resection	16	48.5
Pulmonary lobectomy	17	51.5

**Table 2:** Comparison of APC/KRAS/TP53 mutations in primary tumors and pulmonary metastases.

Patient	APC		KRAS		TP53	
	Primary	*P*M	Primary	*P*M	Primary	*P*M
1	S1415Rfs^*∗*^4	S1415Rfs^*∗*^4	G12D	G12D	I255S	I255S
2	Q358Afs^*∗*^6/R564^*∗*^	Q358Afs^*∗*^6/R564^*∗*^	—	—	F109 C	F109 C
3	—	—	—	—	Splice site	Splice site
4	Q767^*∗*^/S1495Ifs^*∗*^12	Q767^*∗*^/S1495Ifs^*∗*^12	G12D	G12D	G266 V	G266 V
5	E1353^*∗*^	E1353^*∗*^	G12 R	G12 R	P278S	P278S
6	R213^*∗*^/Q1303^*∗*^	Q1303^*∗*^	G12 V	—	T125 T	T125 T
7	T1556Nfs^*∗*^3/E984^*∗*^	E984^*∗*^	G12D	G12D	V143 M	V143 M
8	L292Ffs^*∗*^4/E1286Kfs^*∗*^2	L292Ffs^*∗*^4/E1286Kfs^*∗*^2	—	Q61H	E286 K	E286 K
9	S1198^*∗*^/I1401Cfs^*∗*^7	S1198^*∗*^/I1401Cfs^*∗*^7	—	—	R248 W	R248 W
10	T1556Nfs^*∗*^3	—	—	—	C242Y	—

## Data Availability

The data used to support the findings of this study are included in the article.
